# Pulmonary Sequestration in Adults: Endovascular and Hybrid Treatment Strategies—A Systematic Review

**DOI:** 10.3390/jcm14217493

**Published:** 2025-10-23

**Authors:** Fanni Éva Szablics, Ákos Bérczi, Balázs Bence Nyárády, Márton Philippovich, Ádám Szőnyi, Edit Dósa

**Affiliations:** Department of Interventional Radiology, Heart and Vascular Center, Semmelweis University, 1122 Budapest, Hungaryberczi.akos@semmelweis.hu (Á.B.);

**Keywords:** pulmonary sequestration, intralobar sequestration, extralobar sequestration, endovascular intervention, embolization, stent-graft, hybrid treatment

## Abstract

**Background and Objectives**: Pulmonary sequestration (PS) is a rare congenital lung malformation. In adults, intralobar disease with recurrent infection or hemoptysis predominates. Cross-sectional imaging (CTA/MRA) is central to mapping the aberrant systemic supply; catheter angiography is used when noninvasive imaging is inconclusive or when an endovascular procedure is planned. We aimed to synthesize adult PS cases treated with endovascular or hybrid approaches and to summarize case selection, techniques, and outcomes. **Methods**: We conducted a PRISMA-2020-informed systematic review. We searched PubMed and Scopus from 1 January 2000 to 31 May 2025. Two reviewers extracted data independently; due to heterogeneity, we performed a narrative synthesis and a JBI-adapted qualitative risk-of-bias appraisal. Eligible studies enrolled adults (≥18 years) with imaging-confirmed PS treated with embolization, stent-graft exclusion, or hybrid therapy; prespecified outcomes included technical and clinical success, complications, recurrence, and re-intervention. The review was not registered. **Results**: Of 93 records screened, 41 publications reporting 48 adults were included. Twenty-five patients were managed endovascularly and 23 with hybrid therapy. Intralobar sequestration predominated (36/48); feeding arteries most often arose from the descending thoracic aorta (28/48). Complications were reported in 10 cases, mostly minor; three embolization cases required re-intervention. **Conclusions**: Endovascular therapy is useful for selected anatomies and urgent bleeding control, while hybrid strategies may benefit large, complex, or aneurysmal feeding arteries. The evidence base is limited to small case reports/series with heterogeneous outcome definitions and follow-up, precluding quantitative synthesis. Standardized outcome definitions, structured follow-up, and prospective registries are needed.

## 1. Introduction

Pulmonary sequestration (PS) is a congenital anomaly of the lower respiratory tract characterized by dysplastic, nonfunctioning lung tissue that lacks communication with the tracheobronchial tree and is supplied by aberrant systemic arteries [1,2]. Although PS is the second most common congenital lung anomaly, it remains rare overall, representing roughly 0.15–6.4% of congenital pulmonary malformations at birth, with population-level prevalence estimates of approximately 0.15–1.8% and probable under-recognition in mild disease [3,4,5,6]. In adults, intralobar sequestration (ILS) predominates, while extralobar sequestration (ELS) is less common [2,5,7]. Key clinicopathologic distinctions between ILS and ELS that influence diagnosis and management are summarized in Table 1.

Accurate delineation of arterial supply and venous drainage is central to diagnosis and treatment planning. Computed tomography angiography (CTA) is the principal modality for anatomic mapping; magnetic resonance angiography is used in selected contexts, and digital subtraction angiography (DSA) is reserved when noninvasive imaging is inconclusive or when an endovascular procedure is planned [1,5,8,9]. Resection remains definitive therapy in many adults and is frequently achieved via minimally invasive approaches when anatomy permits [10,11,12,13,14,15]. Endovascular strategies expand options for bleeding control, downstaging before surgery, or, in selected small, simple anatomies and in high-risk surgical candidates, definitive treatment [14,16,17,18,19,20,21,22,23,24]. To provide a coherent synthesis for adult care, this systematic review collates 93 screened records and 41 included publications reporting 48 adult cases, integrating patient and lesion features with procedural details and outcomes.

**Table 1:** Principal distinctions between intralobar and extralobar sequestration relevant to adult management [1,2,3,4,5,6,8,25].

Characteristic	Intralobar Sequestration	Extralobar Sequestration
Pleural investment	Shares the visceral pleura with adjacent lung (no separate pleural covering)	Has its own, separate pleural covering
Venous drainage	Usually to pulmonary veins	Often to systemic veins (azygos, hemiazygos, inferior vena cava)
Typical location	Lower lobes; posterobasal segments; left predominance	Lower lobes; variable extrapulmonary attachments
Systemic arterial supply	Descending thoracic or abdominal aorta and branches	Similar origins; may involve diaphragmatic or abdominal branches
Associated congenital anomalies	Less frequent (~10–15%)	More frequent (~50–65%)
Clinical presentation	Recurrent infections, cough, chest/back pain, hemoptysis; may be incidental	Often neonatal/infantile symptoms; adult presentation less common
Age at diagnosis	Adolescence/adulthood common	Neonatal/infancy predominant; occasional later diagnosis
Side predominance	Left more common	Left somewhat more common
Risk of infection	Higher due to shared pleura and retained secretions	Lower; pleural separation limits contamination

### Research Question (PICOS)

We sought to synthesize adult PS cases treated with endovascular or hybrid approaches and to summarize selection criteria, techniques, and outcomes. PICOS framework. Population: adults (≥18 years) with imaging-confirmed PS. Interventions: endovascular embolization (coils, vascular plugs, particles, or liquid agents), stent-graft exclusion, or hybrid strategies (endovascular therapy plus surgical resection). Comparator: not required (rare condition and predominance of single-arm designs). Outcomes: technical success (immediate angiographic or CTA confirmation of complete exclusion of aberrant systemic inflow), clinical success (durable resolution or improvement of index symptoms without unplanned re-intervention over follow-up), complications (minor/major), recurrence, re-intervention, imaging-based lesion evolution, and follow-up duration. Study design: case reports/series/cohorts with an endovascular or hybrid arm.

## 2. Materials and Methods

### 2.1. Protocol and Registration

The review followed PRISMA-2020 reporting principles. The study was not prospectively registered because the evidence base originated from a precompiled corpus assembled during preliminary scoping. For transparency, prespecified protocol elements and any amendments were recorded in an internal protocol; the full document is on file with the authors and available upon reasonable request. To mitigate selection bias, we executed full, reproducible searches in PubMed and Scopus (1 January 2000–31 May 2025), deduplicated records, applied a priori eligibility criteria with dual independent screening and data extraction, and maintained an audit trail of inclusion/exclusion decisions. Full database search strategies are provided in Supplementary Methods S1, and reasons for title/abstract screening exclusions are summarized in Supplementary Table S1; there were no full-text exclusions.

### 2.2. Eligibility Criteria and Outcomes

We included English-language human reports describing adults aged ≥18 years with intralobar or extralobar PS confirmed by imaging and, where available, intraoperative findings. Eligible interventions comprised endovascular embolization using coils, vascular plugs, particles, or liquid agents; aortic stent-graft exclusion of aberrant arterial origins; and hybrid strategies combining endovascular therapy with surgical resection. Comparators were not required owing to the rarity of adult PS and the predominance of single-arm designs. Prespecified outcomes included technical success (immediate angiographic or CTA confirmation of complete exclusion of aberrant systemic inflow), clinical success (durable resolution or improvement of index symptoms without unplanned re-intervention over follow-up), complications (minor/major), recurrence (imaging or clinical relapse), re-intervention (any unplanned endovascular or surgical procedure after the index treatment), and follow-up duration, including imaging-based lesion evolution (Table 2).

### 2.3. Information Sources and Search Strategy

**Figure 1:**
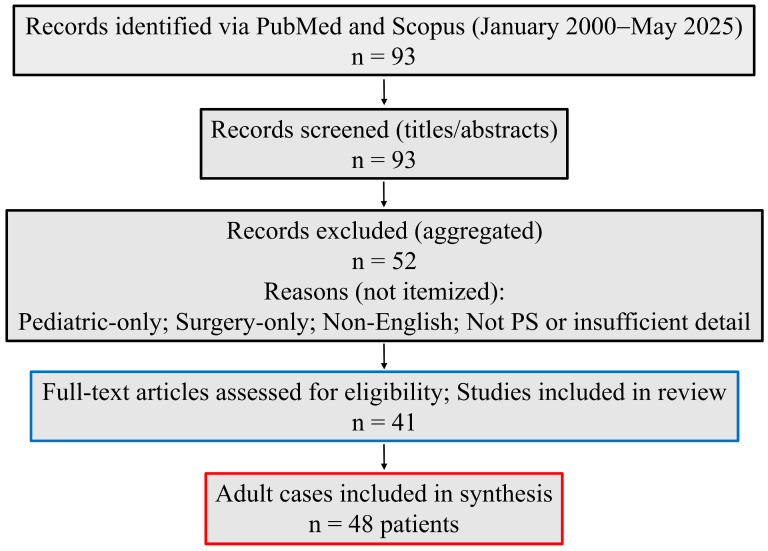
PRISMA-style flow diagram. Records identified via PubMed and Scopus (January 2000–May 2025) (n = 93); records excluded at title/abstract screening (n = 52; pediatric-only; surgery-only; non-English; not PS or insufficient detail); full-text articles included (n = 41 publications). Adult cases included in synthesis: n = 48 patients. No full-text exclusions. PS, Pulmonary sequestration.

We searched PubMed and Scopus for articles published from 1 January 2000, through 31 May 2025, using MeSH terms (PubMed) and free-text keywords related to PS, adults, endovascular treatment, embolization, endografts, stent-grafts, and hybrid procedures. We identified 93 records and applied systematic screening and data extraction (Figure 1). A completed PRISMA-2020 checklist (Checklist S1) and the PRISMA for Abstracts checklist (Checklist S2) are available in the Supplementary Materials; reasons for title/abstract screening exclusions are summarized in Supplementary Table S1. Full database search strategies (PubMed and Scopus) are provided in Supplementary Methods S1.

### 2.4. Study Selection and Data Collection

Two reviewers independently screened titles, abstracts, and full texts, resolving disagreements by consensus. Reasons for exclusion were recorded qualitatively and most commonly reflected pediatric-only cohorts, surgery-only management, non-English publications, and insufficient procedural or outcome detail. A piloted extraction form captured demographics, PS type and location, arterial supply, imaging modalities, endovascular and surgical techniques, complications, recurrence, re-intervention, follow-up duration, and imaging-based lesion evolution. Missing information was documented as not reported without imputation. No automation tools were used for study selection or data extraction; study authors were not contacted.

### 2.5. Risk-of-Bias Assessment

Given the predominance of case reports and small series, we conducted a qualitative risk-of-bias appraisal adapted from the JBI Critical Appraisal tools for case reports/series, using five domains: (i) case definition (adequacy and consistency with accepted criteria); (ii) diagnostic ascertainment (imaging and, where applicable, intraoperative confirmation); (iii) intervention description (sufficient procedural detail to permit replication); (iv) outcome ascertainment (clarity and consistency of technical/clinical success and complications); and (v) follow-up adequacy (duration and imaging/clinical surveillance). Two reviewers judged each domain as Yes (adequate/low concern), No (inadequate/high concern), or NR (not reported/unclear), resolving disagreements by consensus. We present a per-study domain matrix in Supplementary Table S2 and an aggregate domain-level summary in Table 3 (PRISMA-2020 Item 18). We avoided study-level summary scores and did not pool comparative effect measures, in keeping with the heterogeneous, single-arm evidence base.

### 2.6. Synthesis Methods

We performed a narrative synthesis structured by intervention type (endovascular vs. hybrid) and by anatomic features (e.g., feeding artery origin and diameter, PS subtype, presence of aneurysmal feeders, urgency/bleeding). For each synthesis, studies were eligible if they reported the relevant outcome with a clear denominator for descriptive aggregation. No meta-analysis was attempted due to clinical/methodological heterogeneity, small sample sizes, and the absence of comparable effect measures across single-arm designs. No data transformations or imputations were undertaken; missing items were coded as not reported. Continuous variables were summarized as reported (means ± SD); categorical results were presented as descriptive numerators and denominators. Results were tabulated and summarized descriptively; no synthesis software was used. No sensitivity analyses were planned or feasible given the evidence base.

### 2.7. Reporting Bias Assessment

Risk-of-bias due to missing results (reporting bias) was assessed qualitatively. Because all included evidence comprised single-arm case reports or small series with heterogeneous outcomes and follow-up, statistical assessments of small-study effects (e.g., funnel plots, Egger’s test, selection models) were not feasible. Instead, we recorded qualitative signals of potential reporting bias (e.g., non-consecutive inclusion or unclear case ascertainment, selective outcome reporting, incomplete/short follow-up, and non-reporting of technical failures or adverse events) and discussed them narratively in the Discussion. The presence and magnitude of any reporting bias could not be quantified in this evidence base.

## 3. Results

### 3.1. Risk-of-Bias Summary

Reporting was heterogeneous across case-based reports, particularly for explicit outcome definitions and standardized follow-up horizons. Overall, most reports provided clear case definitions and diagnostic imaging, whereas outcome ascertainment and follow-up adequacy were variably documented. The distribution of domain-level judgments is summarized in Table 3, and per-study assessments are provided in Supplementary Table S2. These considerations informed our choice of a narrative synthesis with descriptive numerators and denominators rather than inferential statistics.

### 3.2. Study Selection

From 93 records screened, 41 publications met inclusion and collectively described 48 adult cases treated with endovascular or hybrid strategies [20,21,22,23,24,26,27,28,29,30,31,32,33,34,35,36,37,38,39,40,41,42,43,44,45,46,47,48,49,50,51,52,53,54,55,56,57,58,59,60,61] (Figure 1). Exclusions mainly reflected pediatric-only cohorts, surgical-only reports lacking an endovascular or hybrid component, non-English publications, and studies with inadequate procedural or outcome detail.

### 3.3. Patient Characteristics and Clinical Presentation

Of the 48 cases, sex was male in 29, female in 18, and not reported in one; the mean (±SD) age was 44.9 ± 14.9 years (range, 18–76 years). Most patients were symptomatic (44/48), with hemoptysis the most frequent symptom (22/48, 45.8%), followed by chest, back, or abdominal pain (18/48, 37.5%) and recurrent infections (15/48, 31.3%). Two cases were asymptomatic [42,43] and two lacked symptom details [40,52]. CTA was used in 47/48 cases, with adjunctive DSA in 27/48 [20,21,22,23,26,28,29,30,33,34,35,36,37,38,39,41,42,43,45,49,51,52,54,57] (Table 4).

### 3.4. Anatomy, Lesion Characteristics, and Arterial Supply

Intralobar sequestration was the predominant type (36/48), with ELS confirmed in two cases [35,41]; the subtype was unspecified in the remainder (10/48). The left lower lobe was most commonly involved (24/48), followed by the right lower lobe (19/48). The aberrant systemic artery most frequently originated from the descending thoracic aorta (28/48), with additional origins from the celiac trunk (8/48) and abdominal aorta (7/48). Other arterial sources included the renal and inferior phrenic arteries [26,31,55]; aneurysmal feeding arteries were described in 12 cases [21,34,40,42,44,52,53,54,55,56,57,61] (Table 5).

### 3.5. Interventions and Outcomes

Twenty-five patients underwent endovascular treatment [20,21,22,23,24,26,28,29,30,33,34,35,36,37,38,39,40,41,42,43,44,45] and 23 received hybrid therapy [26,27,31,32,46,47,48,49,50,51,52,53,54,55,56,57,58,59,60,61]. Endovascular approaches included embolization alone (n = 21), stent-graft implantation alone (n = 2), and a combined approach (n = 2). Hybrid care most commonly comprised embolization followed by surgery; video-assisted thoracoscopic surgery (VATS) was favored when feasible [27,46,47,48,50,53,54,58,60,61], and lobectomy was the predominant resection type [27,46,49,51,52,54,55,56,57,58,59,60,61] (Table 6).

Across 39 embolization events (endovascular and hybrid settings combined), coils and microcoils were most frequently used [20,21,22,24,26,27,28,29,30,31,32,34,35,36,39,40,42,43,44,47,48,49,50,51,53,54], followed by vascular plugs [24,29,34,37,41,45,46,47,52] and polyvinyl alcohol (PVA) particles [20,21,26,30,33,38,39,43]. Liquid agents such as N-butyl cyanoacrylate were reported in selected cases, and gelatin sponge was also employed [20,40,45,50]. Stent-graft exclusion was typically chosen for large or aneurysmal feeding arteries or when the ostium was amenable to aortic coverage [23,42,55,56,57,61].

Technical success (immediate exclusion of aberrant systemic inflow) was not consistently reported; clinical recovery was common, although formal definitions and follow-up horizons varied. Complications occurred in 10 cases, predominantly minor. Three embolization cases required re-intervention [29,39,44] (Table 6).

## 4. Discussion

### 4.1. Summary of Evidence

This review consolidates adult experiences with endovascular and hybrid management of PS and places them in the context of the largely surgical literature. Three patterns emerged. First, ILS predominates in adults and most often involves the left lower lobe; aberrant systemic inflow typically arises from the descending thoracic aorta, consistent with historic compilations and contemporary imaging overviews [1,5,8,25]. Second, symptomatic presentation is common (hemoptysis, recurrent infections, and chest or back pain predominate), yet clinical severity, anatomic complexity, and comorbidities vary widely [10,11,15,17]. Third, while resection remains definitive treatment in many centers (often via minimally invasive approaches), endovascular and hybrid strategies have assumed key roles for anatomies in which embolization or stent-graft exclusion can provide hemostasis, reduce arterial inflow (downstage vascularity), or, in selected small/simple lesions, serve as definitive therapy [10,11,12,13,14,17,20,21,22,23,24,46].

### 4.2. Clinical Implications

Clinical decision-making is therefore anatomy- and symptom-driven. Smaller lesions supplied by a single, nonaneurysmal systemic artery with a favorable take-off and course are plausible candidates for definitive embolization, provided that postprocedural imaging confirms complete exclusion and serial follow-up documents progressive involution [14,17,28,29,30,31,32,33,35,36]. In contrast, larger or complex lesions; multiple feeding arteries; short ostial necks at the aorta (limited proximal landing zones); or aneurysmal change favor a planned hybrid pathway (devascularization followed by resection) both to reduce intraoperative bleeding and to improve operative visualization [31,32,33,46,47,48,49,50,51,52,53,54,55,56,57,58,59,60,61]. Nonoperative candidates with persistent symptoms can benefit from palliative endovascular control with symptom-guided surveillance.

### 4.3. Technique Selection

Technique selection is dictated by vascular geometry and flow dynamics. Coils and microcoils are versatile in small, tortuous, or branching feeding arteries and allow a distal-to-proximal strategy; vascular plugs provide rapid, secure occlusion in larger or straighter channels and can buttress coil constructs; and particles (e.g., PVA) achieve more distal penetration but require caution in high-flow beds [21,29,34,35,36,41,47,48,49,50,51,52,59]. Aneurysmal dilation of aberrant systemic arteries appears relatively frequent in adult cohorts undergoing endovascular or hybrid therapy, likely reflecting chronic exposure of pulmonary-type vessels to systemic pressures, adverse take-off angles with turbulent flow, and a publication bias toward more complex cases [42,62]. In such settings, and for feeding arteries with aortic ostia, stent-graft exclusion of the ostium (often combined with distal embolization to prevent retrograde perfusion) may offer durable vascular control, mitigate rupture risk, and serve as a bridge or adjunct to surgery while facilitating subsequent resection if needed [23,24,55,56,57].

### 4.4. Timing Within Hybrid Care

Timing within hybrid care is individualized. Same-session or short-interval surgery (hours to a few days) may minimize the risk of recanalization, limit infectious complications in chronically inflamed parenchyma, and capitalize on diminished arterial inflow to reduce blood loss [31,32,57]. Longer intervals of weeks can be reasonable for clinical stabilization, optimization, or complex planning, although adhesions or collateralization may accrue and complicate resection [51,55]. These trade-offs emphasize the value of multidisciplinary planning.

### 4.5. Complications and Re-Intervention

**Figure 2:**
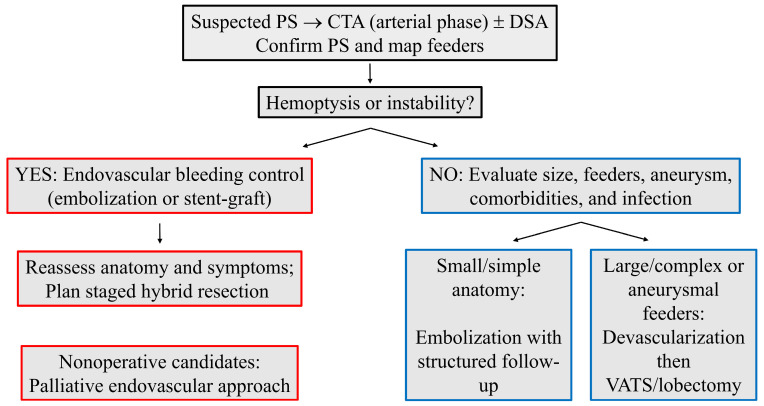
Clinical decision pathway for adult PS. After CTA (arterial phase) ± DSA to confirm PS and map systemic feeding arteries, urgent hemoptysis or instability prompts endovascular bleeding control (embolization or aortic stent-graft), followed by reassessment and planned staged hybrid resection; nonoperative candidates may receive palliative endovascular therapy. In stable patients, small/simple anatomy favors embolization with structured imaging follow-up, whereas large/complex or aneurysmal feeding arteries favor hybrid devascularization followed by VATS/lobectomy. CTA, Computed tomography angiography; DSA, digital subtraction angiography; PS, pulmonary sequestration; VATS, video-assisted thoracoscopic surgery.

Across adult reports, complications following endovascular therapy are mostly minor and self-limited (postembolization pain, fever, and pleuritic symptoms) and typically resolve within about two weeks with supportive care [2,5,17,20,21,22,23,38,39]. Re-intervention, when required, usually reflects residual branches, collateral recruitment, device migration, or recanalization [29,39,44]. Several lesion and vessel features appear to coincide with higher procedural complexity and postprocedural events: (i) large sequestration size and chronic parenchymal inflammation; (ii) multiple or short-neck systemic feeding arteries; (iii) aneurysmal dilation and high-flow dynamics; and (iv) active infection at presentation or recurrent hemoptysis requiring urgent control. These observations, while hypothesis-generating and limited by case report designs, support an anatomy-based selection framework favoring hybrid devascularization plus resection for large, complex, or aneurysmal feeding arteries and reserving definitive embolization for small, single-feeder, nonaneurysmal anatomies with reliable follow-up imaging (Figure 2).

### 4.6. Practice Implications

Given the heterogeneity and reporting gaps across source studies, the following practice implications should be interpreted as descriptive signals rather than comparative effect estimates.

We recommend explicit definitions for technical success (angiographic or CTA exclusion of all systemic inflows), clinical success (durable resolution or improvement of index symptoms without unplanned re-intervention), and recurrence (imaging or clinical relapse requiring further treatment), accompanied by a structured imaging schedule at 1, 3, 6, and 12 months for nonresected lesions [14,17]. Patient selection can be operationalized by anatomy and physiology: definitive embolization for small, single-feeder, nonaneurysmal lesions in suitable candidates; hybrid devascularization plus VATS or limited thoracotomy for large, complex, or aneurysmal feeding arteries; and palliative endovascular control for nonoperative patients, with attention to infection control and symptom-guided follow-up [10,12,13,14,23,24,30,31,32,45,55,56,57].

### 4.7. Limitations

The evidence base is constrained by heterogeneity in design and reporting. Most source studies are single cases or small series with nonstandardized definitions of technical and clinical success, inconsistent imaging protocols, incomplete outcome reporting, and widely variable follow-up horizons, increasing susceptibility to selection and detection biases. In addition, because the dataset originated from a precompiled corpus, residual selection and reporting biases may persist despite deduplication, dual independent screening/extraction, and publication of full search strings. We therefore refrained from quantitative synthesis and instead presented explicit numerators and denominators, treating percentages as descriptive signals rather than estimates of comparative effect sizes.

### 4.8. Future Work

Future work should prioritize multicenter registries and prospective observational cohorts with harmonized outcome definitions, minimum imaging datasets, and lesion-level descriptors (type, size, feeder diameter and tortuosity, aneurysmal change, and venous drainage). Carefully adjusted comparative designs are needed to refine selection thresholds among endovascular, hybrid, and surgical pathways and to examine optimal timing within hybrid care. Agreement on a core outcome set (including standardized definitions for technical and clinical success, recurrence, and imaging-based involution) would substantially improve interpretability across studies.

## 5. Conclusions

Endovascular therapy is an important component of adult PS care (critical for urgent hemostasis and, in anatomically simple lesions, a potential definitive strategy), while hybrid approaches appear advantageous for large, complex, or aneurysmal feeding arteries by improving operative safety. Standardized outcome definitions and structured imaging follow-up, along with prospective registries, are essential to refine selection, timing, and durability.

## Figures and Tables

**Table 2:** Eligibility criteria, outcomes, and exclusions used in this review.

Domain	Definition
Population	Adults (≥18 years) with intralobar or extralobar PS confirmed by imaging and, where available, intraoperative findings.
Interventions	Endovascular embolization (coils, vascular plugs, particles, or liquid agents); aortic stent-graft exclusion of aberrant arterial origins; or hybrid strategies combining endovascular therapy with surgical resection.
Comparators	Not required; single-arm reports included due to rarity and design constraints.
Outcomes	Technical success, clinical success, complications, recurrence, re-intervention, imaging-based lesion evolution, and follow-up duration.
Study designs	English-language human case reports, case series, and cohorts including an endovascular or hybrid arm.
Exclusions	Pediatric-only reports; surgery-only reports without an endovascular or hybrid component; non-PS lesions; non-English publications; insufficient detail to classify intervention or outcomes; duplicate reports merged where identified.

PS, Pulmonary sequestration.

**Table 3:** Risk-of-bias summary by domain (JBI-adapted; percentages calculated as n/41 studies).

Domain	Yes, n (%)	No, n (%)	NR, n (%)
Case definition	38 (92.7)	3 (7.3)	0 (0)
Diagnostic ascertainment	40 (97.6)	1 (2.4)	0 (0)
Intervention description	39 (95.1)	2 (4.9)	0 (0)
Outcome ascertainment	36 (87.8)	5 (12.2)	0 (0)
Follow-up adequacy	23 (56.1)	7 (17.1)	11 (26.8)

Codes: Yes = clearly described and appropriate (lower concern); No = inadequately described or inappropriate (higher concern); NR = not reported/unclear. Per-study domain matrix: see Supplementary Table S2.

**Table 4:** Patient characteristics and clinical presentation across included cases and treatment subgroups.

Characteristic	All (n = 48)	Endovascular Treatment (n = 25)			Hybrid Treatment (n = 23)	
		Embolization (n = 21)	Stent-Graft (n = 2)	Embolization + Stent-Graft (n = 2)	Surgery + Embolization (n = 16)	Surgery + Stent-Graft (n = 7)
Sex (male/female/not reported)	29/18/1	11/9/1	2/0/0	2/0/0	7/9/0	7/0/0
Age (years), mean ± SD	44.9 ± 14.9	40 ± 14.6	36.5 ± 0.7	39 ± 5.7	47.3 ± 14.8	58.1 ± 11
Symptomatic, n (%)	44 (91.7)	19 (90.5)	2 (100)	1 (50)	15 (93.8)	7 (100)
Hemoptysis, n (%)	22 (45.8)	15 (71.4)	0 (0)	1 (50)	5 (31.3)	1 (14.3)
Chest, back, or abdominal pain, n (%)	18 (37.5)	7 (33.3)	1 (50)	1 (50)	6 (37.5)	3 (42.9)
Recurrent infections, n (%)	15 (31.3)	7 (33.3)	1 (50)	0 (0)	3 (18.8)	4 (57.1)
Cough, expectoration, n (%)	9 (18.8)	4 (19)	0 (0)	0 (0)	5 (31.3)	0 (0)
Fever, n (%)	7 (14.6)	2 (9.5)	0 (0)	0 (0)	5 (31.3)	0 (0)

Percentages are calculated within each column (denominators as shown in the headers). SD, Standard deviation.

**Table 5:** Lesion characteristics and arterial supply by treatment subgroup.

Characteristic	All (n = 48)	Endovascular Treatment (n = 25)			Hybrid Treatment (n = 23)	
		Embolization (n = 21)	Stent-Graft (n = 2)	Embolization + Stent-Graft (n = 2)	Surgery + Embolization (n = 16)	Surgery + Stent-Graft (n = 7)
Type						
ILS, n (%)	36 (75)	13 (61.9)	0 (0)	2 (100)	15 (93.8)	6 (85.7)
ELS, n (%)	2 (4.2)	2 (9.5)	0 (0)	0 (0)	0 (0)	0 (0)
NR, n (%)	10 (20.8)	6 (28.6)	2 (100)	0 (0)	1 (6.3)	1 (14.3)
Location						
Left lower lobe, n (%)	24 (50)	9 (42.9)	2 (100)	2 (100)	7 (43.8)	4 (57.1)
Right lower lobe, n (%)	19 (39.6)	9 (42.9)	0 (0)	0 (0)	8 (50)	2 (28.6)
Other, n (%)	5 (10.4)	3 (14.3)	0 (0)	0 (0)	1 (6.3)	1 (14.3)
PS maximum diameter (mm), mean ± SD	67.1 ± 31.5 (n = 12)	51.5 ± 36.1 (n = 2)	NR	NR	74.5 ± 33 (n = 8)	53 ± 25.5 (n = 2)
Feeding artery origin						
Thoracic aorta, n (%)	28 (58.3)	14 (66.7)	2 (100)	2 (100)	4 (25)	6 (85.7)
Celiac trunk, n (%)	8 (16.7)	2 (9.5)	0 (0)	0 (0)	6 (37.5)	0 (0)
Abdominal aorta, n (%)	7 (14.6)	3 (14.3)	0 (0)	0 (0)	4 (25)	0 (0)
Other or no data, n (%)	5 (10.4)	2 (9.5)	0 (0)	0 (0)	2 (12.5)	1 (14.3)
Aneurysmal feeding artery, n (%)	12 (25)	4 (19)	0 (0)	1 (50)	3 (18.8)	4 (57.1)
Feeding artery maximum diameter (mm), mean ± SD	30.6 ± 38.5 (n = 21)	26.2 ± 41.8 (n = 9)	NR	27 (n = 1)	37 ± 52.2 (n = 5)	32.3 ± 29.5 (n = 6)

Percentages are calculated within each column (denominators as shown in the headers). For PS maximum diameter and feeding artery maximum diameter, n reflects the number of reports with available data in that column. ELS, Extralobar sequestration; ILS, intralobar sequestration; NR, not reported; PS, pulmonary sequestration; SD, standard deviation.

**Table 6:** Procedural and post-procedural parameters of endovascular and hybrid treatments.

Parameter	All (n = 48)	Endovascular Treatment (n = 25)			Hybrid Treatment (n = 23)	
		Embolization (n = 21)	Stent-Graft (n = 2)	Embolization + Stent-Graft (n = 2)	Surgery + Embolization (n = 16)	Surgery + Stent-Graft (n = 7)
Embolic agents used						
Coils, n (%)	29 (60.4)	15 (71.4)	-	2 (100)	12 (75)	-
Vascular plug, n (%)	12 (25)	6 (28.6)	-	1 (50)	5 (31.3)	-
PVA, n (%)	8 (16.7)	8 (38.1)	-	0 (0)	0 (0)	-
Other agents, n (%)	4 (8.3)	3 (14.3)	-	0 (0)	1 (6.3)	-
Hospitalization (days), mean ± SD	4.1 ± 2.7 (n = 36)	2.9 ± 3 (n = 15)	3 (n = 1)	4 ± 4.2 (n = 2)	5.4 ± 2.1 (n = 13)	4.8 ± 1.9 (n = 5)
Complications, n (%)	10 (20.8)	8 (38.1)	0 (0)	1 (50)	1 (6.3)	0 (0)
Recurrence, n (%)	2 (4.2)	2 (9.5)	0 (0)	0 (0)	0 (0)	0 (0)
Re-intervention, n (%)	3 (6.3)	3 (14.3)	0 (0)	0 (0)	0 (0)	0 (0)
Follow-up (months), mean ± SD	15.3 ± 15.4 (n = 32)	20.8 ± 17.5 (n = 18)	7.5 ± 6.4 (n = 2)	0.5 ± 0.7 (n = 2)	8 ± 5.3 (n = 7)	13.7 ± 15 (n = 3)
Imaging-based size reduction, involution, n	9 (of 48)	7 (of 21)	1 (of 2)	1 (of 2)	-	-

Percentages are calculated within each column (denominators as shown in the headers). For length-of-stay and follow-up, n reflects the number of reports with available data in that column. PVA, Polyvinyl alcohol; SD, standard deviation.

## Data Availability

Full database search strategies, a summary of title/abstract screening exclusions by reason, and the per-study risk-of-bias table are provided as Supplementary Files. The full protocol can be obtained from the corresponding author upon reasonable request. No analysis code was generated.

## Supplementary Materials

The following supporting information can be downloaded at: https://www.mdpi.com/article/10.3390/jcm14217493/s1, Table S1: Studies excluded at full-text review with reasons (none); title/abstract screening exclusions by reason (summary); Table S2: Risk-of-bias appraisal (JBI-adapted); Methods S1: Full database search strategies; Checklist S1: PRISMA-2020 checklist; Checklist S2: PRISMA-2020 for abstracts–checklist (narrative systematic review).
