# Supplementary material for: Pulmonary Sequestration in Adults: Endovascular and Hybrid Treatment Strategies—A Systematic Review
Source: J Clin Med. 2025 Oct 23;14(21):7493. doi: 10.3390/jcm14217493 (PMC12608632; doi:10.3390/jcm14217493)
Supplement: Supplementary file 1 [file jcm-14-07493-s001.zip › jcm-3815437-supplementary.pdf]

Supplementary Checklist S1. PRISMA-2020 checklist

Manuscript: Pulmonary Sequestration in Adults: Endovascular and Hybrid Treatment Strategies – A Systematic Review

This checklist maps each PRISMA-2020 item to where it is reported in the manuscript (section/subsection or figure), with brief notes on applicability.

**Section and topic**	**Item**	**PRISMA-2020 item**	**Where reported**	**Notes**
TITLE	1	Identify the report as a systematic review.	Title page; Abstract	Title uses 'Systematic Review'; Abstract Methods states systematic review.
ABSTRACT	2	See the PRISMA-2020 for abstracts checklist.	Abstract	Separate PRISMA-2020 abstract checklist provided.
INTRODUCTION	3	Rationale: describe the rationale for the review in the context of what is already known.	1. Introduction	
INTRODUCTION	4	Objectives: provide an explicit statement of the objectives or questions being addressed.	1.1. Research Question (PICOS)	PICOS paragraph inserted.
METHODS	5	Eligibility criteria: specify inclusion/exclusion criteria and how studies were grouped for synthesis.	2.2. Eligibility Criteria and Outcomes; Table of criteria	
METHODS	6	Information sources: specify all databases, registers, and other sources searched, with dates.	2.3. Information Sources and Search Strategy; Suppl. Methods S1 (full strings)	PubMed and Scopus; window 2000–2025.
METHODS	7	Search strategy: present full search strategies for all databases, including filters and limits used.	Suppl. Methods S1	Full PubMed/Scopus strings included.
METHODS	8	Selection process: specify methods used to decide whether a study met the inclusion criteria.	2.4. Study Selection and Data Collection	Dual independent screening; consensus. No automation tools used.
METHODS	9	Data collection process: describe methods of data extraction from reports.	2.4.	Piloted form; duplicate extraction. Authors were not contacted.
METHODS	10a	Data items: list and define all outcomes for which data were sought.	2.2.	Technical/clinical success, complications, recurrence, imaging-based lesion evolution, follow-up.
METHODS	10b	Data items: list and define other variables for which data were sought.	2.2.; 2.4. Extraction fields	Demographics, PS type/location, feeder origin/size, modality.

METHODS	11	Study risk-of-bias assessment: specify methods used to assess risk-of-bias of included studies.	2.5. Risk-of-Bias Assessment	Qualitative appraisal adapted from JBI domains.
METHODS	12	Effect measures: specify the effect measures used for each outcome.	2.5.	Descriptive statistics only; no comparative effect measures; no meta-analysis.
METHODS	13a	Synthesis methods: describe processes used to decide which studies were eligible for each synthesis.	2.2.–2.5.; 3.1.–3.5. (Results subsections)	Single-arm designs; grouped by endovascular vs hybrid.
METHODS	13b	Methods to prepare data and combine results; handling of missing data.	2.5.; 3.1.–3.5.	No imputation; missing marked as not reported.
METHODS	13c	Methods to tabulate or visually display results of individual studies and syntheses.	Tables 2–6; Figure 1	Descriptive tables; PRISMA flow.
METHODS	13d	Methods used to synthesize results.	2.5.; 2.6. Synthesis Methods; 3.1.–3.5.	Narrative synthesis; no meta-analysis performed; no synthesis software used; rationale provided (heterogeneity, small case series).
METHODS	13e	Methods to explore heterogeneity (e.g., subgroup analysis).	2.5.; 2.6.	Narrative exploration by anatomy and approach; no quantitative heterogeneity analysis.
METHODS	13f	Sensitivity analyses.	2.6.	Not applicable (no meta-analysis).
METHODS	14	Reporting bias assessment: describe methods used to assess risk-of-bias due to missing results.	2.5.; 2.7. Reporting Bias Assessment; 4. Discussion	Publication bias discussed qualitatively; no statistical assessment (due to small, heterogeneous single-arm case series).
METHODS	15	Certainty assessment: describe any methods used to assess certainty (e.g., GRADE).	2.5.; 4.	GRADE not performed due to case report/small series evidence without comparative effect estimates; certainty discussed narratively.
RESULTS	16a	Study selection: describe results of the search and selection, ideally with a flow diagram.	3.2. Study Selection; Figure 1 (PRISMA)	93 records screened; 41 included.
RESULTS	16b	Cite studies that might appear to meet the inclusion criteria, but	—	No full-text exclusions; all 41 assessed were included; 52 excluded at title/abstract stage.

		were excluded, and explain why.		
RESULTS	17	Study characteristics: present characteristics for which data were extracted.	3.2.–3.5.; Tables 4–5	
RESULTS	18	Risk-of-bias in studies: present assessments for each included study.	2.5.; 3.1. Risk-of-Bias Summary; Table 3; Suppl. Table S2	Per-study JBI-adapted domain judgements (Yes/No/NR) by two independent reviewers; consensus resolution; no summary scores.
RESULTS	19	Results of individual studies.	3.2.–3.5.; Tables 2–6	Summarized in descriptive tables; no effect sizes.
RESULTS	20	Results of syntheses.	3.1.–3.5.; 4.	Narrative synthesis; no meta-analysis.
RESULTS	21	Reporting biases.	3.1.; 4.	Publication/reporting bias considered qualitatively.
RESULTS	22	Certainty of evidence.	2.7.; 3.1.; 4.	Discussed narratively; no formal GRADE.
DISCUSSION	23a	Provide a general interpretation of the results in the context of other evidence.	4.	
DISCUSSION	23b	Discuss limitations of the evidence included in the review.	4.	Case report design, heterogeneity, incomplete reporting.
DISCUSSION	23c	Discuss limitations of the review processes used.	2.1. Protocol and Registration; 4.	No registration; structured yet limited dataset.
DISCUSSION	23d	Implications for practice, policy, and future research.	4.; 5. Conclusion	Selection framework; need for registries and standardized outcomes.
OTHER INFORMATION	24a	Registration information.	2.1.	Not prospectively registered; rationale provided.
OTHER INFORMATION	24b	Protocol access.	2.1.	No publicly accessible protocol.
OTHER INFORMATION	25	Support (including funding), role of funders.	Funding statement	The funder had no role in study design, data collection, analysis, interpretation, or manuscript writing.
OTHER INFORMATION	26	Competing interests.	Conflicts of Interest	
OTHER INFORMATION	27	Availability of data, code, and other materials.	Data Availability Statement; Suppl. Methods S1; Suppl. Table S1; Suppl. Table S2	Full search strings; excluded studies; per-study risk-of-bias table; no analysis code generated.

NR, Not reported; *PS*, pulmonary sequestration.

Supplementary Checklist S2. PRISMA-2020 for abstracts – checklist (narrative systematic review)

Section	Item	Where reported in abstract
Title	Identify the report as a systematic review.	Title includes "– A Systematic Review".
Background	Brief background/context.	Background: Pulmonary sequestration (PS) is a rare congenital lung malformation. In adults, intralobar disease with recurrent infection or hemoptysis predominates.
Objectives	Explicit statement of objectives.	Objectives: To synthesize adult PS cases treated with endovascular or hybrid approaches and to summarize case selection, techniques, and outcomes.
Data sources	Information sources and date of last search.	Methods: We searched PubMed and Scopus from 1 January 2000 to 31 May 2025.
Eligibility criteria	Key inclusion criteria and prespecified outcomes.	Eligible studies enrolled adults (≥18 years) with imaging-confirmed PS treated with embolization, stent-graft exclusion, or hybrid therapy; prespecified outcomes included technical and clinical success, complications, recurrence, and re-intervention.
Participants and interventions	Key participants and interventions.	Adults (≥18 years) with imaging-confirmed PS; interventions included embolization, stent-graft exclusion, and hybrid strategies.
Study appraisal and synthesis	Methods of appraisal and synthesis.	We conducted a PRISMA-2020-informed systematic review... narrative synthesis and a JBI-adapted qualitative risk-of-bias appraisal.
Results	Included studies and key findings.	Results: Of 93 records screened, 41 publications reporting 48 adults were included... Complications were reported in 10 cases, mostly minor; three embolization cases required re-intervention.
Limitations	Primary limitations of evidence.	Limitations: The evidence consists of small case reports/series with heterogeneous outcome definitions and follow-up; quantitative synthesis was not feasible.
Conclusions	General interpretation and implications.	Conclusions: Endovascular therapy is useful for selected anatomies and urgent bleeding control, while hybrid strategies may benefit large, complex, or aneurysmal feeding arteries. Standardized outcome definitions, structured follow-up, and prospective registries are needed.
Registration	Registration number (or statement).	Not registered.

PS, Pulmonary sequestration.

Supplementary Methods S1. Full database search strategies

The exact database queries used in this review are provided below to support reproducibility.

PubMed search string (example):

PubMed (searched 31 May 2025):

(("Pulmonary Sequestration"[MeSH Terms] OR "pulmonary sequestration"[Title/Abstract] OR "bronchopulmonary sequestration"[Title/Abstract]) AND (adult[MeSH Terms] OR adult*[Title/Abstract] OR "18 years"[Title/Abstract]) AND (endovascular[Title/Abstract] OR emboli*[Title/Abstract] OR "stent-graft"[Title/Abstract] OR endograft*[Title/Abstract] OR TEVAR[Title/Abstract] OR hybrid[Title/Abstract])) AND ("2000/01/01"[Date - Publication]: "2025/05/31"[Date - Publication])

Filters: Humans; English.

Scopus search string (example):

Scopus (searched 31 May 2025):

TITLE-ABS-KEY (("pulmonary sequestration" OR "bronchopulmonary sequestration") AND (adult OR adults) AND (endovascular OR embolization OR embolisation OR "stent-graft" OR endograft OR TEVAR OR hybrid)) AND PUBYEAR > 1999 AND PUBYEAR < 2026 AND (LIMIT-TO(LANGUAGE, "English"))

Supplementary Table S1. Studies excluded at full-text review with reasons (none); title/abstract screening exclusions by reason (summary)

According to the PRISMA flow for this review, all full-text articles assessed for eligibility (n = 41) were included; there were no full-text exclusions. Records excluded at title/abstract screening (n = 52) are summarized below by reason to enhance transparency. Reason categories were mutually exclusive and applied in a prespecified hierarchy (as listed).

Title/abstract screening exclusions by reason

Reason category	Definition	Count (n)	Proportion (%)
Pediatric-only cohort	Participants <18 years or mixed cohorts without an extractable adult subgroup	6	11.5
Surgery-only management	No endovascular or hybrid component reported	32	61.5
Non-English article	Language ineligible (non-English)	3	5.8
Insufficient detail	Insufficient procedural or outcome detail in the title/abstract to determine eligibility	2	3.8
Non-PS lesion or ineligible population	Study not about PS in adults	9	17.3
Total		52	100

PS, Pulmonary sequestration.

Supplementary Table S2. Risk-of-bias appraisal (JBI-adapted)

Coding: Yes/No/NR. Two independent reviewers performed domain-level judgements; disagreements were resolved by consensus. No summary score was calculated. Domain definitions are provided below.

**Study (year)**	**Case definition**	**Diagnostic ascertainment**	**Intervention description**	**Outcome ascertainment**	**Follow-up adequacy**
Chataut et al. (2024)	Yes	Yes	Yes	Yes	No
Marine et al. (2022)	Yes	Yes	Yes	Yes	Yes
Deng et al. (2022)	Yes	Yes	Yes	Yes	Yes
He et al. (2020)	Yes	Yes	Yes	Yes	Yes
Healy et al. (2019)	Yes	Yes	Yes	Yes	No
Ellis et al. (2018)	Yes	Yes	Yes	Yes	Yes
Borzelli et al. (2018)	Yes	Yes	Yes	Yes	Yes
Zener et al. (2017)	Yes	Yes	Yes	Yes	Yes
Tartaglia et al. (2016)	Yes	Yes	Yes	No	No
Ojha et al. (2015)	Yes	Yes	Yes	Yes	Yes
Kim et al. (2014)	Yes	Yes	Yes	No	No
Mohapatra et al. (2012)	Yes	Yes	Yes	Yes	Yes
Greben et al. (2012)	No	No	Yes	Yes	Yes
Marine et al. (2011)	Yes	Yes	Yes	Yes	Yes
Leoncini et al. (2011)	Yes	Yes	Yes	Yes	No
Türk et al. (2025)	Yes	Yes	Yes	No	Yes
Hordijk et al. (2025)	Yes	Yes	Yes	Yes	Yes
Kim et al. (2012)	Yes	Yes	Yes	Yes	Yes
Madhusudhan et al. (2009)	Yes	Yes	Yes	Yes	Yes
Chen et al. (2019)	Yes	Yes	Yes	Yes	Yes
Nemoto et al. (2020)	Yes	Yes	Yes	Yes	Yes
Wilder et al. (2019)	Yes	Yes	Yes	Yes	No
Grossi et al. (2022)	Yes	Yes	Yes	No	NR
Wang et al. (2021)	Yes	Yes	Yes	Yes	No
Hakiri et al. (2021)	Yes	Yes	Yes	Yes	Yes
Zhou et al. (2018)	Yes	Yes	Yes	Yes	NR
Sakai et al. (2018)	Yes	Yes	Yes	Yes	Yes
Petty et al. (2017)	Yes	Yes	No	No	NR

Fabbri et al. (2018)	Yes	Yes	Yes	Yes	Yes
Ferland et al. (2015)	Yes	Yes	No	Yes	NR
Goto et al. (2013)	Yes	Yes	Yes	Yes	Yes
Ragusa et al. (2010)	No	Yes	Yes	Yes	NR
Nahal et al. (2024)	Yes	Yes	Yes	Yes	NR
Monfregola et al. (2024)	Yes	Yes	Yes	Yes	NR
Yamamoto et al. (2020)	Yes	Yes	Yes	Yes	NR
Sridhar et al. (2019)	Yes	Yes	Yes	Yes	Yes
Hewett et al. (2016)	Yes	Yes	Yes	Yes	NR
Yamasaki et al. (2014)	Yes	Yes	Yes	Yes	NR
Nakagiri et al. (2010)	Yes	Yes	Yes	Yes	Yes
Porez et al. (2025)	No	Yes	Yes	Yes	NR
Fukuyama et al. (2023)	Yes	Yes	Yes	Yes	Yes

NR, Not reported.

Domain definitions (applied across single-arm case reports/series):
• **Case definition:** Pulmonary sequestration clearly identified (e.g., systemic arterial supply on CTA/MRA ± pathology).
• **Diagnostic ascertainment:** Objective diagnostic evidence presented (imaging ± pathology).
• **Intervention description:** Endovascular/hybrid intervention described with sufficient detail to reproduce (device/technique/approach).
• **Outcome ascertainment:** Technical/clinical outcomes assessed using defined, objective criteria (e.g., angiographic/CTA confirmation, prespecified success/failure definitions).
• **Follow-up adequacy:** Adequate follow-up per protocol (e.g., ≥3–6 months clinical ± at least one imaging control).
